# Intrinsically robust and scalable biofilm segmentation under diverse physical growth conditions

**DOI:** 10.1016/j.isci.2024.111386

**Published:** 2024-11-13

**Authors:** Jian-geng Chiou, Todd Kwang-Tao Chou, Jordi Garcia-Ojalvo, Gürol M. Süel

**Affiliations:** 1 Department of Molecular Biology, School of Biological Sciences, University of California San Diego, La Jolla, CA 92093 USA; 2 Department of Medicine and Life Sciences, Universitat Pompeu Fabra, 08003 Barcelona, Spain

**Keywords:** Natural sciences, Biological sciences, Microbiology

## Abstract

Developmental patterning is a shared feature across biological systems ranging from vertebrates to bacterial biofilms. While vertebrate patterning benefits from well-controlled homeostatic environments, bacterial biofilms can grow in diverse physical contexts. What mechanisms provide developmental robustness under diverse environments remains an open question. We show that a native clock-and-wavefront mechanism robustly segments biofilms in both solid-air and solid-liquid interfaces. Biofilms grown under these distinct physical conditions differ 4-fold in size yet exhibit robust segmentation. The segmentation pattern scaled with biofilm growth rate in a mathematically predictable manner independent of habitat conditions. We show that scaling arises from the coupling between wavefront speed and biofilm growth rate. In contrast to the complexity of scaling mechanisms in vertebrates, our data suggests that the minimal bacterial clock-and-wavefront mechanism is intrinsically robust and scales in real time. Consequently, bacterial biofilms robustly segment under diverse conditions without requiring cell-to-cell signaling to track system size.

## Highlights

• Developmental patterning of biofilms is robust to growth conditions
• Developmental patterns of biofilms scale with growth rate
• Scaling is intrinsic to the clock-and-wavefront mechanism
• Scaling does not require cell-cell communication

## Graphical abstract

**Figure:**
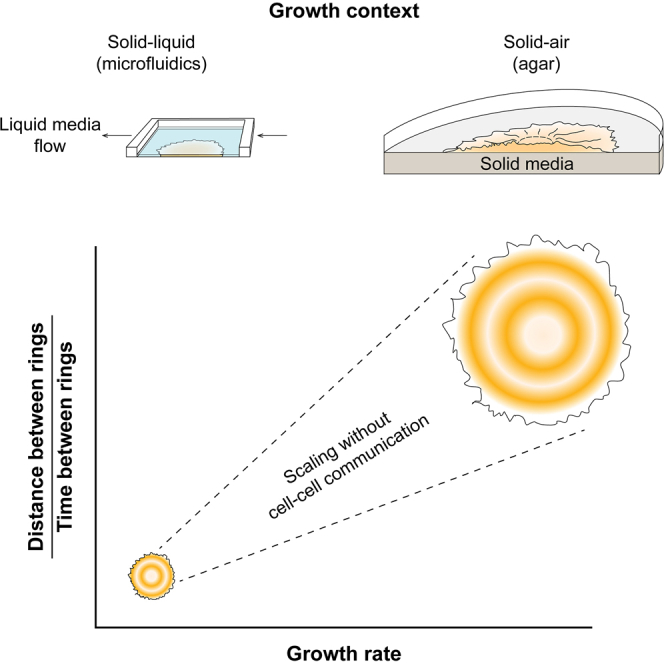

## Introduction

**Figure 1:**
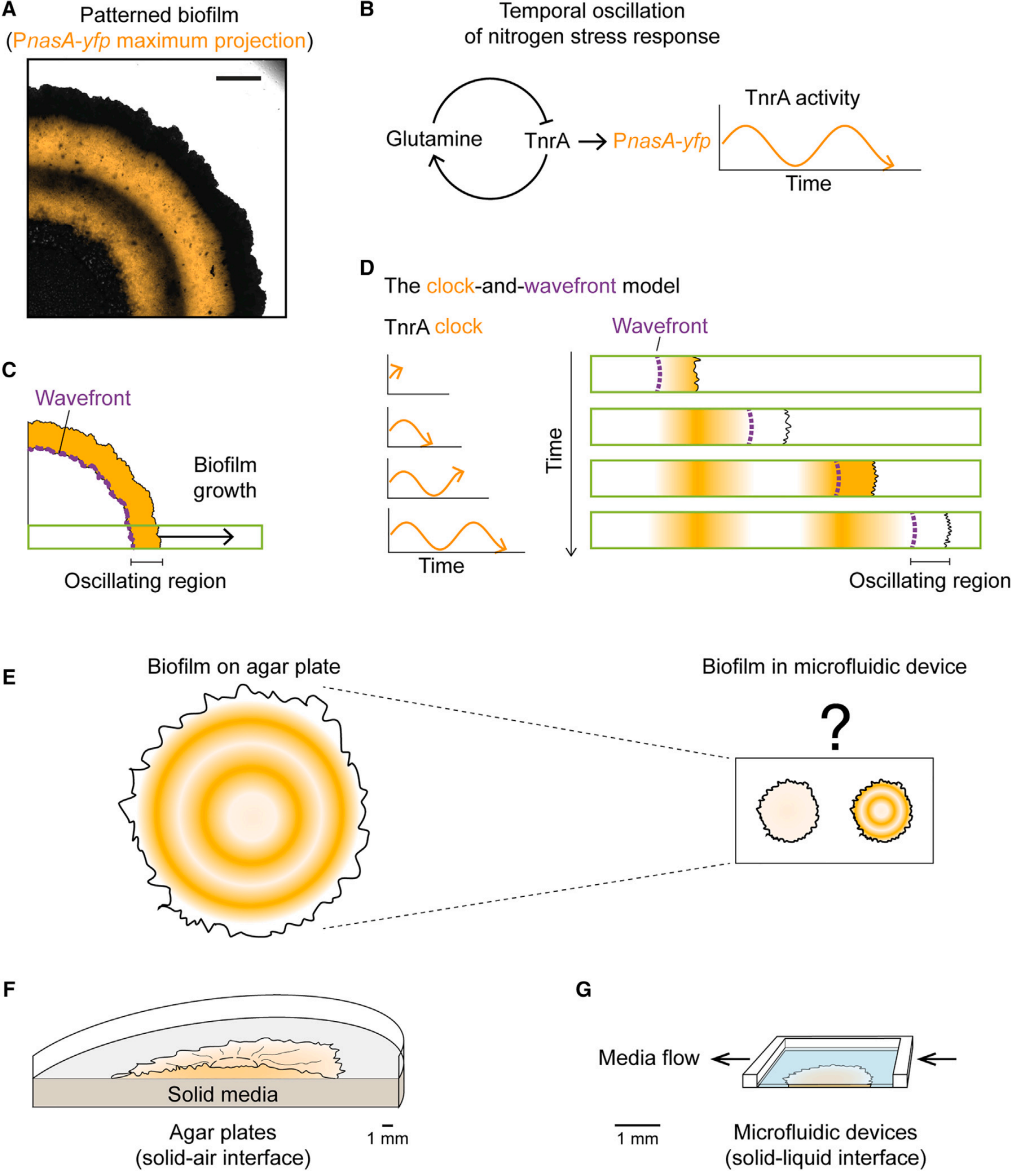
Are patterns in biofilms robust to developmental contexts and community size? (A) *Bacillus subtilis* biofilms grown on agar media have been reported to develop concentric ring patterns in their nitrogen stress response. (Adapted from [12]) Scale bar, 1 mm. (B) The concentric ring pattern is driven by a temporal oscillation using a molecular oscillator of nitrogen stress response. P_*nasA*_-*yfp* fluorescent signal is used to monitor nitrogen stress response. (C and D) A wavefront that marks the edge of the oscillating region sweeps across the biofilm as a biofilm grows. Cells that are left behind the wavefront become metabolically dormant and preserve the previous oscillation phase as repeated spatial patterns. This patterning mechanism is referred to as the clock-and-wavefront mechanism. (E) Do small biofilms generate patterns like large biofilms? It is possible that small biofilms, for example, grown in microfluidic devices, do not reach a size large enough to show any obvious patterns. (F) A schematic of the solid-air interface on agar plates where biofilm patterning has been discovered. (G) The solid-liquid interface in microfluidic devices is an alternative growth condition where biofilms are fully submerged. Arrows indicate the direction of media supply.

Living systems from bacteria to vertebrates must function robustly under different environmental and extracellular constraints. Developmental patterning is an example of such a biological function. Vertebrates promote robust development by placing their embryos under well-controlled homeostatic conditions within eggs or inside a uterus. Furthermore, vertebrate embryos have a tightly regulated and predetermined size.^1^^,^^2^^,^^3^ However, bacterial biofilms are fully exposed to the physical environment in which they grow, and their size could vary drastically depending on the physical habitat.^4^ These distinctions from vertebrates raise the question of whether biofilm patterning can occur robustly under different growth conditions. Previous research on spatial patterning within bacterial biofilms focused on stochastically self-organizing patterns,^5^^,^^6^^,^^7^^,^^8^ without a scaling mechanism, or on patterns generated by nutrient gradients,^9^^,^^10^^,^^11^ where scaling is determined by the environment. Recently, we discovered that *Bacillus subtilis* biofilms can autonomously segment their nitrogen stress response spatially into concentric rings in a deterministic manner (Figure 1A), by translating a temporal oscillation of nitrogen response regulators (Figure 1B) into spatially repeating rings of gene expression^12^ (Figures 1C and 1D). This deterministic patterning process is conceptually similar to the clock-and-wavefront segmentation mechanism that patterns vertebrate somitogenesis,^13^ but takes place in bacterial biofilms where neither their habitats nor system size is pre-determined. It remains unclear whether a simple clock-and-wavefront mechanism can robustly generate patterns under diverse developmental contexts, such as solid-air^14^^,^^15^ or solid-liquid interfaces.^16^^,^^17^^,^^18^^,^^19^

We compared *B. subtilis* biofilms grown on standard solid agar pads (Figure 1F)^12^ to those grown in a microfluidic device, submerged in liquid media with constant flow (Figure 1G). Biofilms within the microfluidic device grew at a slower rate and reached a substantially smaller size^19^ compared to their counterparts on agar plates. The difference in length scales raised the question of whether such a small biofilm could generate any obvious segmentation pattern (Figure 1E). Despite the difference in growth context and sizes, we find that biofilms in the microfluidic device also form segmentation patterns, similar to biofilms grown on agar. Developmental patterning is thus a robust feature of *B. subtilis* biofilms, arising under two starkly distinct physical growth conditions. Moreover, quantitative measurements of segmentation patterns in both conditions reveal a simple mathematical relationship, consistent with scaling to biofilm growth rate by the clock-and-wavefront mechanism.

The ability of a cellular population to scale often requires that cells locally know their positions relative to the size of the population using gradients of diffusible morphogens.^20^^,^^21^ Quorum sensing molecules are diffusible signals that could build such a gradient during biofilm development.^14^^,^^22^ However, our results indicate that the bacterial clock-and-wavefront mechanism is sufficient to scale developmental patterning without the need for quorum sensing to communicate colony size. Scaling of biofilm patterning is thus intrinsic to the patterning mechanism, making biofilm segmentation robust to colony size and growth context.

## Results

### Biofilms develop robust patterns under different growth contexts

**Figure 2:**
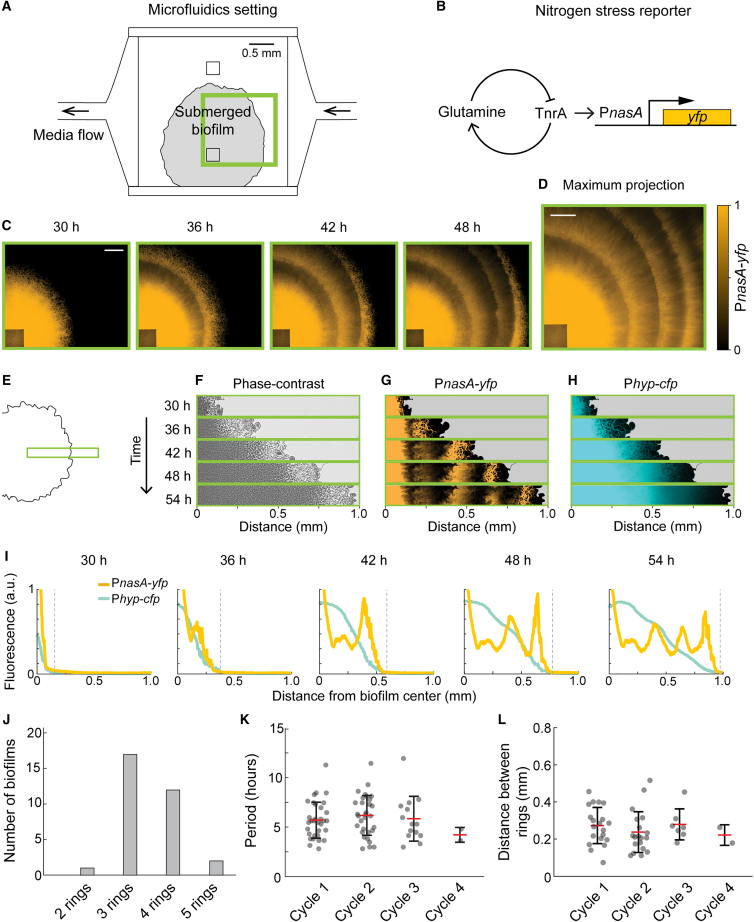
Small biofilms in microfluidic devices generate concentric ring segments (A) A schematic showing the microfluidic setup. The microfluidic chamber is 3 mm × 3 mm in length and width and 6 μm in height. Microscope field of view indicated by the green rectangle is shown in later (C) and (D). Two cell traps at the center of the chamber allows planktonic cells to be loaded into the microfluidic chamber. We purged cells at the top cell trap to initiate only one biofilm from the bottom cell trap. Due to the asymmetric localization of the biofilm, biofilm growth is anisotropic because media supply at the bottom side of the biofilm is eventually blocked as biofilms grow. (B) Nitrogen stress response is reported using a fluorescent transcriptional reporter driven by the *nasA* promoter directly regulated by TnrA. (C) Timelapse images of the P_*nasA*_*-yfp* reporter during biofilm development as the biofilm expands from the cell trap. Timestamps indicate hours after planktonic cells are loaded under the cell trap. Scale bar, 0.2 mm. (D) Maximum projection of the 54 h timelapse of the same movie as (C). (C) and (D) share the same normalized color scale. Scale bar, 0.2 mm. (E–H) Timelapse images of a biofilm corresponding to the field of view as indicated in (A). The biofilm contains both a P_*nasA*_-*yfp* reporter and a P_*hyperspank*_-*cfp* (P*hyp*-*cfp*) reporter. P*hy*p-*cfp* was constitutively induced by 1 mM IPTG and serves as a control for fluorescence measurements and cell density. Regions outside of the biofilm edge were cropped out according to phase-contrast images in (E). Timestamps indicate hours after planktonic cells are loaded under the cell trap. (I) Fluorescence intensity profiles of (D) and (E), background-subtracted and normalized for each reporter separately. Gray dashed lines indicate the edge of the biofilm determined using (C). (J) A summary of the number of rings per biofilm generated in *n* = 32 biofilms. (K) The distribution of the temporal period in each oscillation cycle, defined as the time between the occurrence of two consecutive rings. Bars indicate the mean and standard deviation. (L) Distribution of distance between rings in each P_*nasA*_-yfp oscillation cycle, measured using the local maxima of P_*nasA-yfp*_ in the maximum projection image of the entire timelapse movie. Bars indicate the mean and the standard deviation.

To compare the formation of *B. subtilis* biofilms in two physically distinct environmental conditions, we grew them on solid agar media (solid-air interface) and in a microfluidic device (solid-liquid interface) (Figure 2A). In both cases, biofilms were supplied with the same defined media.^12^ However, the microfluidic chamber is 3 mm × 3 mm wide with a height of 6 μm, allowing biofilm expansion only in the horizontal direction. In addition, nutrients are only provided to the periphery of the biofilm through constant liquid media flow. This contrasts with biofilms grown on solid agar plates, where nutrients are available from underneath the biofilm, and where biofilms can expand in both the horizontal and vertical directions. To directly compare the effects of the physical differences between these two growth environments, we used the same defined media in both the microfluidic device and in agar plates.^12^ We monitored biofilm development within the microfluidic device using time-lapse microscopy. Specifically, we quantified the spatial and temporal patterning of the nitrogen stress response by using a previously characterized P_*nasA*_-*yfp* transcriptional fluorescent reporter^12^ (Figure 2B). The *nasA* promoter is upregulated by TnrA,^23^ which is the major transcription factor that regulates the *B. subtilis* nitrogen stress response.^24^^,^^25^ To control for non-specific transcriptional dynamics during biofilm growth, the cells also contained a second reporter, P_*hyperspank*_-*cfp*, which was constitutively expressed with 1 mM isopropyl β-*d*-1-thiogalactopyranoside (IPTG) in the media. This approach allowed us to quantitatively track biofilm development within the microfluidic device and measure gene expression as a function of space and time.

We tracked biofilms grown inside microfluidic devices for up to 72 h and observed concentric ring patterns of P_*nasA*_-*yfp* expression that were qualitatively similar to those reported for biofilms grown on agar plates (Figures 2C, 2D and Video S1, compared to Figure 1A). Specifically, around 36 h after loading, the biofilm began to spatially segment such that P_*nasA*_-*yfp* expression formed multiple concentric rings with a period of around 6 h (5.9 ± 0.23 mean ± SEM) (Figures 2E–2G, 2I and Video S2). The P_*nasA*_-*yfp* patterns are slightly anisotropic due to the asymmetric localization of the biofilm (Figures 2C and 2D). As the biofilm grows, the side closest to the chamber wall will press up against it, limiting media flow and growth. This limitation introduces a slight anisotropy in both biofilm expansion and the concentric rings of P_*nasA*_-*yfp* expression.

Video S1. Ring pattern in microfluidic biofilm, related to Figure 2c Timelapse movie of a biofilm containing the P_*nasA*_*-yfp* reporter as the biofilm expand from the cell trap. YFP signal is color-coded orange and overlaid phase-contrast images are color-coded in gray. Timestamps indicate hours:minutes after planktonic cells are loaded under the cell trap. Time points 33:20 and 33:30 were skipped due to media replenishment. Scale bar, 100 μm

Video S2. Ring pattern with constitutive reporter and phase contrast in microfluidic biofilm, related to Figure 2 Timelapse three-color movie of a biofilm containing a P_*nasA*_*-yfp* reporter (orange) and a P_*hyperspank*_*-cfp* reporter (cyan). The P_*hyperspank*_*-cfp* reporter was under full induction at 1 mM IPTG. Phase-contrast images are in gray. The YFP and CFP movies are overlaid with the corresponding phase-contrast movies to highlight the edge of the biofilm. Timestamps indicate hours:minutes after planktonic cells were loaded under the cell trap. Scale bar, 100 μm

We observed that biofilm segmentation was specific to the nitrogen response as measured by P_*nasA*_ expression. No such patterning was visible with the constitutively expressed P_*hypserspank*_-*cfp* control reporter, whose expression monotonically decreased toward the biofilm edge (Figures 2H and 2I). Image analysis showed that biofilms in the microfluidic device typically generated 3–4 rings before running out of space within the microfluidic chamber (Figure 2J). These multiple concentric rings allowed us to document the time and location of each ring at its peak intensity. We then calculated the time intervals (period) and distance between consecutive rings (Figures 2K and 2L). We found that the overall concentric ring patterns formed by biofilms in the microfluidic device were qualitatively similar to biofilms grown on agar plates, despite the differences in the two physical growth conditions.

### The same molecular oscillator drives patterning at different spatial scales

Next, we investigated whether the concentric ring patterns in the two growth contexts were generated by the same molecular clock-and-wavefront mechanism. As reported in the literature and discussed previously, concentric rings formed in biofilms growing on agar plates are driven by a temporal oscillation of the nitrogen stress response^12^ (Figure 1B). These dynamics arise in response to nitrogen limitation by a negative feedback loop mediated by the TnrA transcription factor, which is inhibited by glutamine.^26^ When cells experience glutamine limitation, TnrA becomes active and inhibits the *gltA/B* promoter.^27^ The *gltA/B* gene encodes the GOGAT enzyme that recycles glutamine into glutamate.^28^ Inhibition of GOGAT thus counteracts glutamine depletion, thereby completing the negative feedback loop. This negative feedback generates oscillations of TnrA activity, which subsequently drive oscillatory expression of the P_*nasA*_-*yfp* reporter during biofilm development.^12^

**Figure 3:**
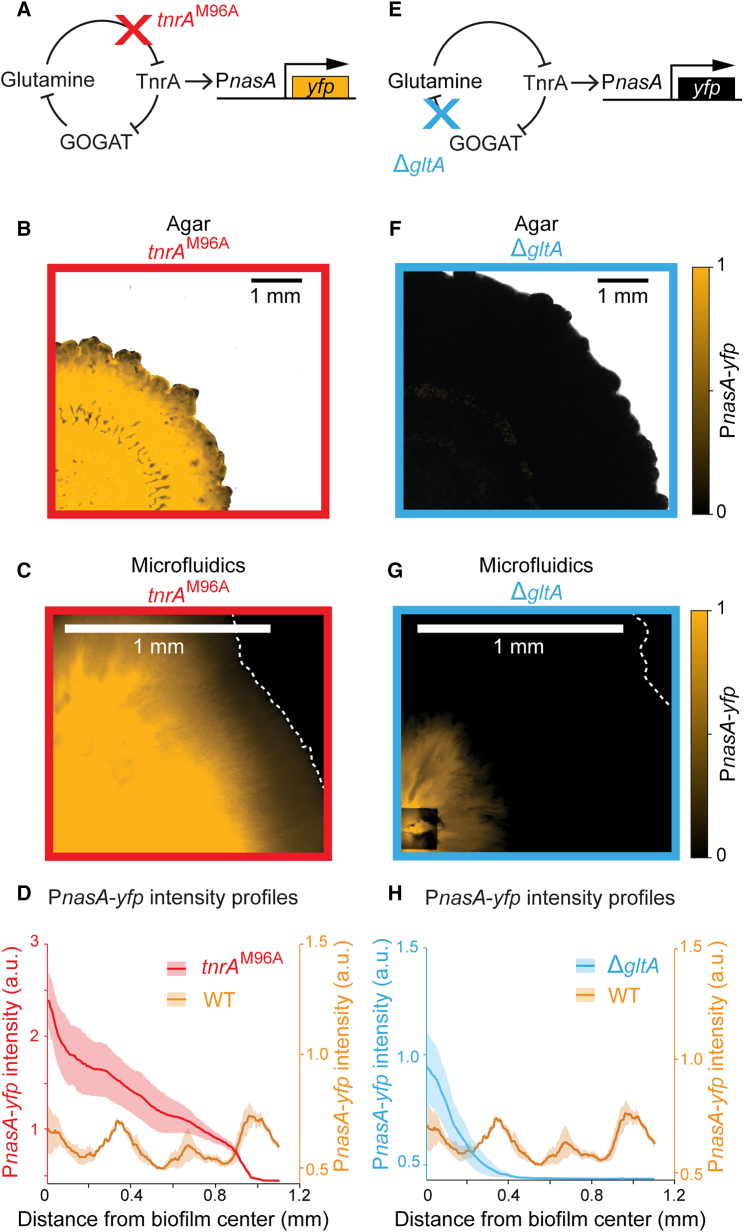
Biofilm patterning in different conditions share the same molecular mechanism (A) A schematic depicting the molecular oscillator. *tnrA*(M96A) has a point mutation on TnrA that interrupts inhibition by glutamine. The oscillator is thus disrupted and TnrA is expected to be constitutively active. (B and C) The maximum projection of the P_*nasA*_-*yfp* timelapse of the *tnrA*(M96A) biofilm grown on agar plates and in the microfluidic device, respectively. Scale bar, 1 mm. (D) P_*nasA*_-*yfp* intensity profile of the maximum projection of *tnrA*(M96A) and wildtype biofilms. Traces show the mean and standard error of three independent biofilms in each strain. (E) GltA is a subunit of the GOGAT enzyme. The deletion of *gltA* disrupts the enzymatic reaction that consumes glutamine. The oscillator is thus expected to be disrupted, and TnrA is expected to stay inactive. (F and G) The maximum projection of the *gltA* deletion biofilm grown on agar plates and in the microfluidic device, respectively. Scale bar, 1 mm. (H)P_*nasA*_-*yfp* intensity profile of the maximum projection of *gltA* deletion and wildtype biofilms. Traces show the mean and standard error of three independent biofilms in each strain.

To determine if the same molecular mechanism drives patterning in biofilms in the microfluidic device, we tested two previously described gene mutations that disrupt P_*nasA*_-*yfp* oscillations in biofilms grown on agar plates.^12^ First, we tested the *tnrA*(M96A) strain, where a point mutation disrupts the ability of TnrA to sense nitrogen limitation^29^ (Figure 3A). This *tnrA*(M96A) strain thus continuously perceives glutamine deficiency and was previously reported to exhibit a constitutively high nitrogen stress response. Consistent with the behavior on agar media, we observed that this mutant strain also formed biofilms in the microfluidic device with persistently high P_*nasA*_-*yfp* expression that lacked a ring pattern^12^ (Figures 3B–3D). Second, we tested the *gltA* deletion mutant strain lacking GOGAT. It was reported that this strain reduces glutamine consumption and relieves nitrogen stress in biofilms grown on agar media (Figure 3E). As expected, biofilms formed by this strain in the microfluidic device also exhibited low P_*nasA*_-*yfp* expression and thus no ring pattern, which is consistent with biofilms formed on agar media (Figures 3G and 3H). These data indicate that the same negative feedback loop underlying the nitrogen stress response drives segmentation for biofilms on agar plates as well as those submerged in the microfluidic device. The same clock-and-wavefront mechanism thus seems to robustly operate under distinct biofilm growth conditions.

### The clock-and-wavefront mechanism scales the pattern based on a predicted mathematical relationship

**Figure 4:**
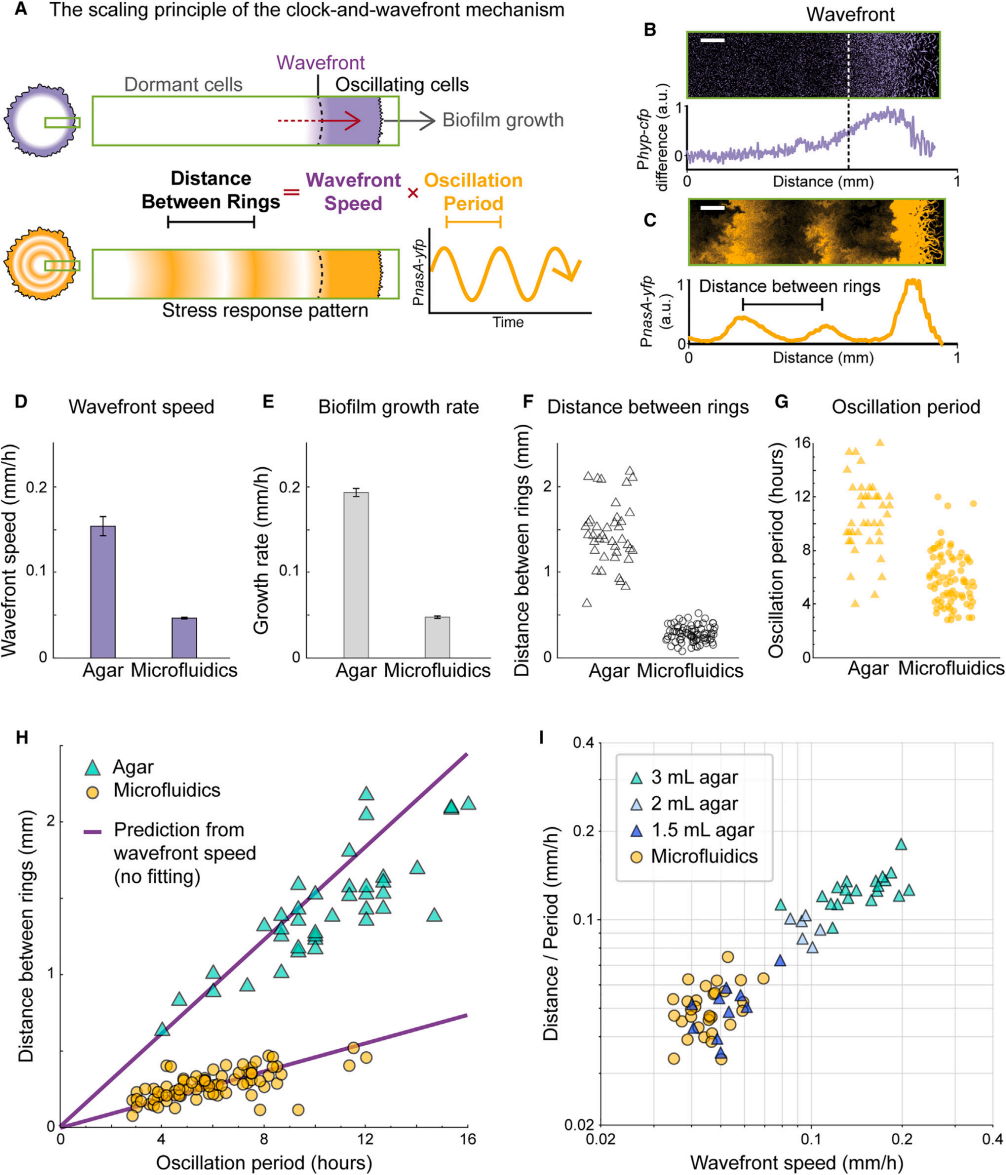
The clock-and-wavefront mechanism intrinsically scales biofilm patterning (A) A schematic illustrating the expected relationship between the wavefront speed, the distance between rings, and the oscillation period, based on the clock-and-wavefront model. The wavefront speed should match the overall biofilm growth rate. (B) P_*hyperspank*_-*cfp* fluorescence intensity difference between consecutive time points. We quantified the intensity profile along the radial axis of the biofilm as indicated in (A), averaged over a 100-pixel width. The position of the wavefront is determined by 50% of the peak intensity. Scale bar, 0.1 mm. (C) P_*nasA*_-*yfp* fluorescence intensity profile of the same region of the same biofilm as (B). Scale bar, 0.1 mm. (D) The average wavefront speed calculated using P_*hyperspank*_-*cfp* difference between the occurrence of consecutive rings. Error bars indicate standard error. *n* = 12 for each growth context. (E) Biofilm growth rates in the two growth contexts measured by the expansion rate of the biofilm edge using phase-contrast images. Error bars indicate standard error. *n* = 32 and 25 for agar and microfluidic biofilms, respectively. (F) The distribution of the distance between P_*nasA*_-*yfp* rings measured in the two growth contexts. (G) The distribution of the P_*nasA*_-*yfp* temporal oscillation period measured in the two growth contexts. (H) A scatterplot showing the relationship between the distance between rings and the oscillation period measured in (F) and (G), compared with the predicted linear relationship based on the mean wavefront speed measured in (D). No data fitting was used. For F and G, *n* = 32 and 79 for agar and microfluidic biofilms, respectively. (I) A scatterplot showing the relationship between the distance between rings divided by the oscillation period and the measured wavefront speed with axes in a log scale. Typical experiments of biofilms grown on agar used 3 mL of media. We decreased the total volume to 2 mL and 1.5 mL, which led to biofilms that correspondingly match the expected relationship defined in (A). *n* = 20 for biofilms at 3 mL agar; *n* = 6 for biofilms at 2 mL agar; *n* = 10 for biofilms at 1.5 mL agar; *n* = 31 for biofilms in the microfluidic device. See also and Figures S1–S3.

We next asked how the same clock-and-wavefront mechanism can generate different length scales of patterning under distinct biofilm growth contexts. We observe that the temporal oscillations in nitrogen stress occur at the actively growing periphery of the biofilm, while the interior of the community becomes metabolically inactive. As the biofilm expands outward, oscillating peripheral cells find themselves shifted toward the interior of the biofilm and cease to oscillate. This leads to a wavefront of metabolic dormancy that moves along with the expanding biofilm edge. This mechanism converts the peripheral temporal oscillations into a persistent spatial pattern within the biofilm (Figure 4A). As previously described,^13^ the clock-and-wavefront mechanism predicts that the distance between rings (S) should be determined by the product of the wavefront speed (v) and the oscillation period (T).

Distancebetweenrings(S)=wavefrontspeed(v)xoscillationperiod(T)

In this way, the clock-and-wavefront mechanism can intrinsically alter the length scale of the concentric ring patterns through variations of either the speed of the wavefront (and thus the rate of growth of the biofilm) or the period of the oscillations, or both. To investigate how these two factors regulate the scaling of the ring pattern, we independently measured the wavefront speed (v), and the distance (S) and oscillation period (T) between consecutive rings.

To quantify the wavefront speed (v) experimentally, we measured the fluorescence intensity of the constitutively expressed P_*hyperspank*_-*cfp* and calculated the change in signal intensity between time frames (Figure 4B). Areas of new fluorescent protein production indicate the metabolically active region of the biofilm. We then traced the position of the edge of this active region over time (Figure S1), thus determining the wavefront speed for both growth conditions (Figure 4D). Similarly, we also traced the position of the biofilm edge over time to determine the overall growth rates of the biofilms. The wavefront speed of biofilms in the microfluidic device was approximately four times slower than that of biofilms grown on agar plates (Figure 4D). The biofilm expansion rate of biofilms within the microfluidic device was also four times slower (Figure 4E). Biofilms in the microfluidic device were thus approximately four times smaller when compared to biofilms grown on agar plates.

To further test the role of the wavefront speed and growth rate in scaling, we utilized the S = vT relationship. In particular, the two different wavefront speeds (v) observed in the microfluidic device and agar plate biofilms predict that each growth context has a different slope in the linear relationship between the distance between consecutive rings and the oscillation period (S/T). To measure the distance between rings (S), we analyzed the dynamics of the P_*nasA*_-*yfp* signal and documented the location and time when each ring formed (Figure 4C). We then determined the distance between two consecutive rings (Figure 4F) and the oscillation period (Figure 4G) for both growth conditions. The measured distance between concentric rings within the biofilm was about four times smaller in the microfluidic device when compared to agar plates. To visualize the S/T ratio, we generated a scatterplot of the distance (S) versus the period (T). (Figure 4H). This plot shows that biofilm data points obtained from either condition overlay closely with the respective prediction based on the independently measured wavefront speeds, without fitted parameters (Figures 4H and S2). In other words, no data fitting was performed to obtain the overlap between the predicted behavior (solid line) and the measured data points. Together, these results demonstrate that the clock-and-wavefront mechanism not only drives robust segmentation, but also underpins the scaling of the concentric ring patterns in biofilms grown under different physical contexts.

To further interrogate the scaling feature of biofilm patterning, we reduced the total agar volume to obtain smaller biofilms. We chose this approach rather than reducing concentrations of nutrients (glutamate and glycerol), since changes in concentration are known to alter the clock period.^12^ We show that simply reducing the total amount of available nutrients results in slower-growing, smaller biofilms. Specifically, reducing the total agar volume from 3 mL to 2 mL and 1.5 mL led to correspondingly smaller biofilms. We then compared all the biofilm patterning data obtained from the microfluidic device and the smaller agar-grown biofilms. Specifically, we plotted the ratio of ring distance/oscillation period against the wavefront speed for every measured biofilm (Figure 4I). Results show that all the biofilm data points collapse into a simple S = vT relationship, regardless of differences in physical growth conditions. Concurrently, the data points obtained from small biofilms grown on limited agar overlapped with data points from biofilms grown in the microfluidic device. This finding demonstrates that biofilms grown in markedly different physical conditions can exhibit quantitatively similar patterning. These data further emphasizes the robustness of the clock-and-wavefront-driven segmentation in biofilms.

### Typical cell-to-cell communication is not required for pattern scaling

**Figure 5:**
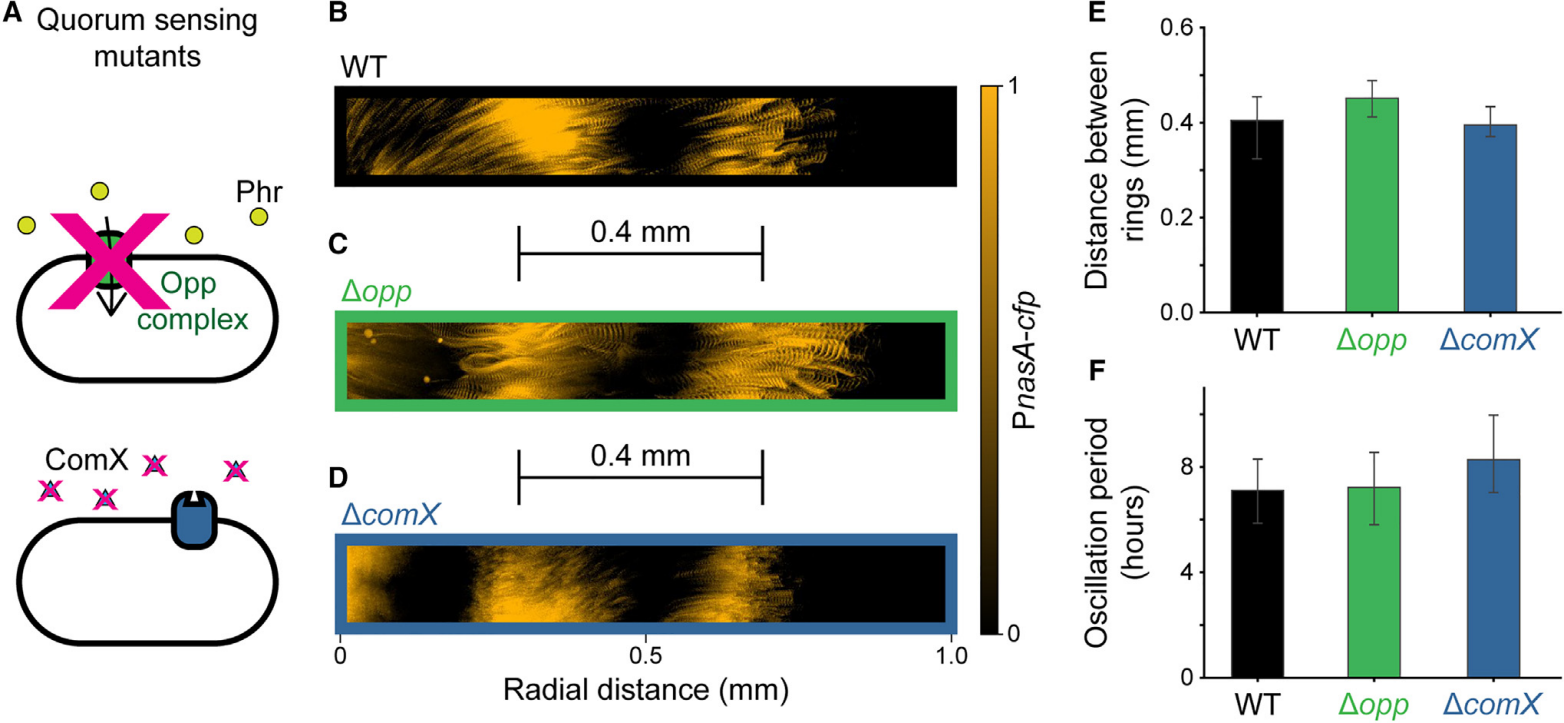
Biofilm patterning does not require cell-to-cell signaling (A) Previous examples of pattern scaling in bacterial communities relied on engineered cell-to-cell communication. However, the clock-and-wavefront mechanism in *B. subtilis* patterning uses a cell-autonomous oscillator, raising the question of whether cell-to-cell communication is required for biofilm pattern scaling. The Rap-Phr system is a major quorum sensing system in *B. subtilis* where the Opp complex imports Phr signal peptides. An *opp* deletion mutant would prevent the import of these communication signals. Another well-characterized cell-to-cell signaling system is the ComQXPA quorum sensing system where ComX acts as a signaling molecule. A *comX* deletion mutant would result in the loss of these signals. (B and C) In biofilms grown in the microfluidic device, an *opp* deletion mutant produced rings similarly to a wildtype biofilm. Images show the maximum intensity projection of P_*nasA*_-*cfp* signal over 48 h after cells were loaded into the microfluidic device. (D) In biofilms grown in the microfluidic device, a *comX* deletion mutant produced rings similarly to a wildtype biofilm. Image shows the maximum intensity projection of P_*nasA*_-*cfp* signal over 48 h after cells were loaded into the microfluidic device. (E) Comparison of the distance between P_*nasA*_-*cfp* rings in *opp* deletion and *comX* deletion biofilms with wildtype biofilms measured on the same day. Rings were separated by similar distances for all three cases. Bars indicate mean and error bars indicate 95% confidence interval. *n* = 3 independent biofilms for wildtype strain; *n* = 6 independent biofilms for *opp* deletion strain; *n* = 3 independent biofilms for *comX* deletion strain. (F) Comparison of the temporal oscillation period between P_*nasA*_-*cfp* rings in *opp* deletion and *comX* deletion biofilms with wildtype biofilms measured on the same day. Rings were separated by a similar temporal period for all three cases. Bars indicate mean and error bars indicate 95% confidence interval. *n* = 3 independent biofilms for wildtype strain; *n* = 6 independent biofilms for *opp* deletion strain; *n* = 3 independent biofilms for *comX* deletion strain.

Finally, we asked whether the surprisingly robust and spatially scaled biofilm patterning was aided by cell-to-cell communication. Quorum sensing molecules may form signal gradients during biofilm development, similar to morphogen gradients in metazoan embryogenesis that could potentially facilitate scaling.^22^^,^^30^ In fact, previous work that achieved scale-invariant patterning in bacterial communities, such as concentric rings of gene expression in *Escherichia coli* communities, relied on engineered cell-to-cell communication.^31^ However, the *B. subtilis* clock-and-wavefront operating in biofilms appears to be a cell-autonomous mechanism.^12^ This raises the question of whether cell-to-cell communication known to exist in bacteria, such as quorum sensing, is also required for robust scaling of biofilm patterning under distinct conditions. We focused on biofilms grown in the microfluidic device as a stringent test, since diffusion of extracellular signals is expected to be higher in liquid media. There are two well-characterized quorum sensing systems that participate in biofilm formation in *B. subtilis.* One of them is the species-specific Rap-Phr system that involves nine short Phr peptides recognized by intracellular Rap receptors.^32^^,^^33^ The import of all extracellular Phr signals is mediated by the *opp* peptide transport complex (Figure 5A). To determine whether this cell-to-cell communication is required to scale the biofilm concentric ring pattern, we tested an *opp* deletion mutant, which is deficient in quorum sensing. Deleting this importer did not affect the formation or scaling of the concentric ring pattern in biofilms grown in the microfluidic device (Figures 5B, 5C, 5E, and 5F). The other *B. subtilis* quorum sensing system is ComQXPA, which is common to all *Bacillus* species.^34^ We deleted *comX*, the signaling peptide in *B. subtilis*, to see if this system was necessary for scaling of patterning. We find that the mutant strain was still able to form biofilm rings when grown in the microfluidic device (Figures 5B, 5D–5F). These results are consistent with previous findings obtained for biofilms grown on agar plates, where adding a diffusion barrier into the biofilm did not affect the formation of the concentric ring pattern.^12^ Our results in Figures 5B–5F show that disrupting known cell-to-cell communication in biofilms^33^^,^^34^ has no impact on scaled pattern formation. Together, these data indicate that cell-to-cell communication is not required to scale the concentric ring pattern of biofilms.

## Discussion

Scalability is an important requirement for biological systems that grow to different sizes but need to self-organize into similar patterns.^35^^,^^36^ The clock-and-wavefront mechanism has been proposed to support such scaling in metazoans, as the length scale of somite patterning depends linearly on the wavefront speed and thus on the growth rate of the system.^13^ However, vertebrate somitogenesis may not be the most canonical example of this elegant mechanism, due to the complex interplay of the underlying signaling components and additional layers of regulation.^37^^,^^38^^,^^39^^,^^40^ Here, we demonstrated that a natural and minimal bacterial clock-and-wavefront mechanism provides intrinsic scalability of developmental patterning in biofilms. We found that qualitatively similar concentric ring patterns of nitrogen stress response arise regardless of growth context or biofilm size. In particular, submerged biofilms in a microfluidic chamber grew up to four times smaller than those on solid agar media, which resulted in an approximately four times slower wavefront speed. Concurrently, we observed that the concentric segmentation pattern of biofilms scaled spatially with wavefront speed. This result confirms the original clock-and-wavefront model expectations, dating back to the 1970s,^13^ regarding its scalability. The slower biofilm growth rate that causes the biofilm to reach a smaller size simultaneously generates a smaller segmentation pattern. Such intrinsic scalability of the clock-and-wavefront mechanism by the growth rate provides a minimal way to scale developmental patterning for bacterial biofilms (Figure 4I).

Our findings also demonstrate the robustness of the bacterial clock-and-wavefront mechanism, which can operate under markedly diverse environmental contexts. While vertebrate development takes place under highly regulated and homeostatic conditions, bacteria must cope with a wide range of physically distinct contexts such as growing on top of a surface or submerged in liquid. The ability to grow under variable habitats is not unique to bacteria, and indeed many organisms including plants, fungi, and protozoa must also cope with diverse physical conditions. Different growth contexts can give rise to communities of varying sizes that may not be knowable *a priori* to the organism. Failure to scale developmental patterns could have biological consequences. If developmental patterns do not conform to biofilm size, a small biofilm may not be able to form spatial patterns, and thus downstream cell differentiation processes such as sporulation may be negatively impacted.^12^^,^^41^ Cell-to-cell communication could in principle help biofilms measure their size. Indeed, synthetic biology approaches have accomplished the scaling of patterns within bacterial communities by using engineered cell-to-cell signaling molecules based on quorum sensing signals.^31^^,^^42^ However, the effectiveness of signaling molecules could be diminished within densely packed biofilms where diffusion-based signaling may be physically impaired,^43^ or such signals may be washed away when the biofilm is submerged in persistent liquid flow. We find that the native bacterial clock-and-wavefront mechanism intrinsically scales developmental patterning based primarily on the biofilm growth rate. In this way, this natural mechanism operates robustly under various environmental conditions without requiring an additional mechanism that communicates overall colony size. The natural clock-and-wavefront mechanism thus provides an elegant and simple solution for scaling developmental patterning in biological systems that can develop under diverse physical growth conditions and reach a highly variable size.

### Limitations of the study

While we were able to alter the growth rate of biofilms growing on agar, changing the growth rate of biofilms in the microfluidic device was more challenging. We used the same nutrient concentrations across all experiments in order to directly compare results from distinct environmental contexts. While altering the flow rate of media could have changed growth rate, we found that lowering the flow rate led to increased clogging of the fluidic channels, and increasing the flow rate increased the risk of cells being washed out of the cell trap. Despite these limitations, the microfluidic setup described here holds promise for future work to investigate, for example, how fluctuating nutrient conditions could affect developmental patterning. Furthermore, we cannot rule out the possibility of additional unknown mechanisms operating during biofilm development. However, our findings indicate that biofilms do not require a collective sensing mechanism to scale their developmental patterns, which challenges the current paradigms.

## Resource availability

### Lead contact

Requests for further information and resources should be directed to and will be fulfilled by the lead contact, Gürol M. Süel (gsuel@ucsd.edu).

### Materials availability

This study did not generate new unique reagents.

### Data and code availability

• All data reported in this paper will be shared by the lead contact upon request.
• This paper does not report original code.
• Any additional information required to reanalyze the data reported in this paper is available from the lead contact upon request.

## Acknowledgments

We acknowledge L. Galera-Laporta and C. Comerci for helpful comments. This work was funded by the following funding sources: 10.13039/100000002National Institutes of Health grant R35GM139645 (GMS), 10.13039/100000002National Institutes of Health grant T32GM127235 (TKC), 10.13039/100000183Army Research Office grants W911NF2410036, W911NF2210107 and W911NF10361 (GMS), 10.13039/100000865Bill & Melinda Gates Foundation INV-067331 (GMS), Spanish Ministry of Science and Innovation project PID2021-127311NB-I00 (JGO), Spanish State Research Agency and FEDER (JGO), and Generalitat de Catalunya ICREA Academia Programme (JGO).

## Author contributions

Conceptualization: J.-g.C., T.K.-T.C., J.G.-O., and G.M.S.; methodology: J.-g.C., T.K.-T.C., J.G.-O., and G.M.S.; investigation: J.-g.C. and T.K.-T.C.; visualization: J.-g.C. and T.K.-T.C.; funding acquisition: J.G.-O. and G.M.S.; project administration: G.M.S.; supervision: G.M.S.; writing—original draft: J.-g.C., T.K.-T.C., J.G.-O., and G.M.S.; writing—review and editing: J.-g.C., T.K.-T.C., J.G.-O., and G.M.S.

## Declaration of interests

The authors declare that they have no competing interests.

## STAR★Methods

### Key resources table

**Table:** 

REAGENT or RESOURCE	SOURCE	IDENTIFIER
**Chemicals, peptides, and recombinant proteins**		

Glycerol	MilliporeSigma	Cat#G5516, CAS: 56-81-5
L-glutamic acid monosodium salt hydrate (anhydrous)	MilliporeSigma	Cat#G5889, CAS: 142-47-2
Magnesium chloride hexahydrate	Fisher Scientific	Cat#BP214, CAS: 7786-30-3
Potassium phosphate monobasic	Fisher Scientific	Cat#BP362, CAS: 7778-77-0
Potassium phosphate dibasic	Fisher Scientific	Cat#BP363, CAS: 7758-11-4
Thiamine HCl	Fisher Scientific	Cat#BP892, CAS: 67-03-8
Manganese chloride	Acros Organics	Cat#AC193451000, CAS: 13446-34-9
Calcium chloride	Fisher Scientific	Cat#BP510, CAS: 10035-04-8
Iron (III) chloride	Acros Organics	Cat#AC217090025, CAS: 10025-77-1
Zinc (II) chloride	MilliporeSigma	Cat#Z0152, CAS: 7646-85-7
Isopropyl β-D-1-thiogalactopyranoside	MilliporeSigma	Cat#IPTG-RO, CAS: 367-93-1
MOPS	MilliporeSigma	Cat#M3183, CAS: 1132-61-2

**Experimental models: Organisms/strains**		

*B. subtilis* NCIB 3610 *amyE::Phyp-cfp, sacA::PnasA-yfp*	Chou et al.^12^	N/A
*B. subtilis* NCIB 3610 *sacA::PnasA-yfp, tnrA::tnrA* M96A	Chou et al.^12^	N/A
*B. subtilis* NCIB 3610 *sacA::PnasA-yfp, gltA::neo*	Chou et al.^12^	N/A
*B. subtilis* NCIB 3610 *amyE::PnasA-cfp*	This paper	N/A
*B. subtilis* NCIB 3610 *oppA-D:: CAT*, *amyE::PnasA-cfp*	This paper	N/A
*B. subtilis* NCIB 3610 *comX:: CAT*, *amyE::PnasA-cfp*	This paper	N/A

**Recombinant DNA**		

ECE174-PnasA-yfp	Michael Elowitz lab, Caltech	N/A
pDL30-PnasA-3xopt-cfp	Michael Elowitz lab, Caltech	N/A

**Software and algorithms**		

Python	Python Software Foundation	https://www.python.org/
R	The R Foundation	https://www.r-project.org/
FIJI	Schindelin et al.^44^	https://fiji.sc/
Radial Profile Extended Plugin	Carl, Philippe	https://imagej.nih.gov/ij/plugins/radial-profile-ext.html

**Other**		

6-well plate	Genesee Scientific	Cat#25-105
CellASIC ONIX B04F Microfluidic Plate	MilliporeSigma	Cat#B04F-01-5PK
Petri dish	Genesee Scientific	Cat#32-107G

### Experimental model and subject details

#### Bacterial strains

All *B. subtilis* strains used in this study are listed in the key resources table. The P_*nasA*_-*yfp* reporter was integrated into the wild-type NCIB3610 strain at the *sacA* locus. The pSac-CM-P_*nasA*_-*yfp* vector used for transformation was a gift from the Michael Elowitz lab (California Institute of Technology, CA). The *opp* operon deletion strain and the *comX* deletion strain were both derived from previous work.^19^ The pDL30-P_*nasA*_-*cfp* vector was a kind gift from the Michael Elowitz lab (California Institute of Technology, CA). Where appropriate, growth media were supplemented with antibiotics at the following concentrations: 5 μg/mL chloramphenicol, 8 μg/mL neomycin, and 300 μg/mL spectinomycin.

### Method details

#### Growth on agar media

Biofilms were grown as previously described.^12^ We used MSgg medium [5 mM potassium phosphate buffer (pH 7.0), 100 mM 3-(N-morpholino)propanesulfonic acid buffer (pH 7.0, adjusted with NaOH), 2 mM MgCl_2_, 700 μM CaCl_2_, 50 μM MnCl_2_, 100 μM FeCl_3_, 1 μM ZnCl_2_, 2 μM thiamine HCl, 0.5% (v/v) glycerol, 0.5% (w/v) monosodium glutamate], previously reported to promote biofilm formation,^45^ supplemented with 1.5% (w/v) agar. We used modified MSgg (3xMSgg) with 1.5% glycerol and 1.5% glutamate, previously used to achieve multiple oscillations of *nasA* expression.^12^ The P_*hyperspank*_ reporter was induced using 1 mM isopropyl β-D-1-thiogalactopyranoside (IPTG). Agar media was poured to a height of 2.5 mm (3 mL). 2 mL and 1.5 mL were used when appropriate. Desired strains were streaked from −80°C glycerol stocks on LB agar plates with appropriate antibiotics and incubated overnight at 37°C. A single colony was inoculated into 2 mL of LB liquid media and incubated with shaking at 37°C for 4.5 h, after which the cultures were normalized according to the optical density (OD) such that all strains had OD of 1.3 in 1.5 mL final volume. Cells were spun down at 2100 rcf for 1.5 min, resuspended in 1.5 mL MSgg, and incubated with shaking at 37°C for 1.5 h for cells to acclimate to MSgg media. 1 μL of cell culture was spotted per agar pad and imaged.

#### Growth in microfluidic device

The day before the experiment, we streaked the desired strains from −80°C glycerol stocks on LB agar plates with appropriate antibiotics. The plates were incubated overnight at 37°C. The following day, a single colony was inoculated into 3 mL LB liquid media and incubated with shaking at 37°C for 5 h. The culture was then spun at 2100 rcf for 1 min, resuspended in fresh 3xMSgg liquid media, and loaded into the cell trap of CellASIC ONIX B04F microfluidic plates following manufacturer protocol (Millipore-Sigma). The microfluidic chambers are 3 mm × 3 mm in length and width. The ceiling is 6μm in height except at the two cell traps where the ceiling is 0.65μm in height. The cells were loaded at 8 psi, where all cells flowed through the microfluidic chamber unless stuck under the cell trap. After loading, we purged the cells stuck under the upper cell trap to initiate biofilm growth only at the bottom cell trap. Cells were incubated at 30°C with 3xMSgg media being supplied at 2 psi, while we observed cells forming biofilms from the cell trap using time-lapse microscopy. The P_*hyperspank*_ reporter was induced using 1 mM IPTG.

#### Time-lapse microscopy

Biofilms on agar media were observed using time-lapse microscopy with an Olympus IX81 microscope with a Lambda XL light source (Sutter Instruments), and a 2.5x objective (Olympus). Images were taken with an ORCA-Flash4.0 V2 camera (Hamamatsu) at 40 min intervals. Biofilms in the microfluidic device were observed using time-lapse microscopy with an Olympus IX83 microscope with an X-Cite Turbo LED light source (Excelitas Technologies), and a 10x objective (Olympus), Images were taken with an ORCA-Flash4.0 LT + camera (Hamamatsu) at 10 min intervals.

### Quantification and statistical analysis

Fiji (https://imagej.nih.gov/ij/, RRID: SCR_002285), Python (https://www.python.org/, RRID: SCR_008394), and R (https://www.r-project.org/, RRID: SCR_001905) were used for image analysis.

Biofilms on agar plates were analyzed as previously described.^12^ Biofilms in microfluidic devices were analyzed in a similar manner. Briefly, we used the Radial Profile Extended plugin in Fiji to find the mean intensities along the radial profile of the biofilm for each image in the movie. The time between *nasA* rings was determined by using the local maxima of the time trace outputted by the Radial Profile Extended plugin. The distance between *nasA* rings was determined by using the maximum projection image. Statistical analysis was performed using R.

The position of the wavefront was determined as previously described.^12^ Briefly, we subtracted the pixel intensities between consecutive time frames of a P_*hyperspank*_-*cfp* movie. We defined the position of the wavefront using 50% of the maximum difference value. The wavefront speed was measured by analyzing the displacement of the wavefront position over time.

Statistical details, including the exact value of *n* and precision measures, can be found in the figure legends where the data appears. This work did not perform measurements of statistical significance.
