# Supplementary material for: Intrinsically robust and scalable biofilm segmentation under diverse physical growth conditions
Source: iScience. 2024 Nov 13;27(12):111386. doi: 10.1016/j.isci.2024.111386 (PMC11635021; doi:10.1016/j.isci.2024.111386)
Supplement: Document S1. Figures S1–S3 [file mmc1.pdf]

iScience, Volume *27*

Supplemental information

Intrinsically robust and scalable biofilm segmentation under diverse physical growth conditions

Jian-geng Chiou, Todd Kwang-Tao Chou, Jordi Garcia-Ojalvo, and Gürol M. Süel

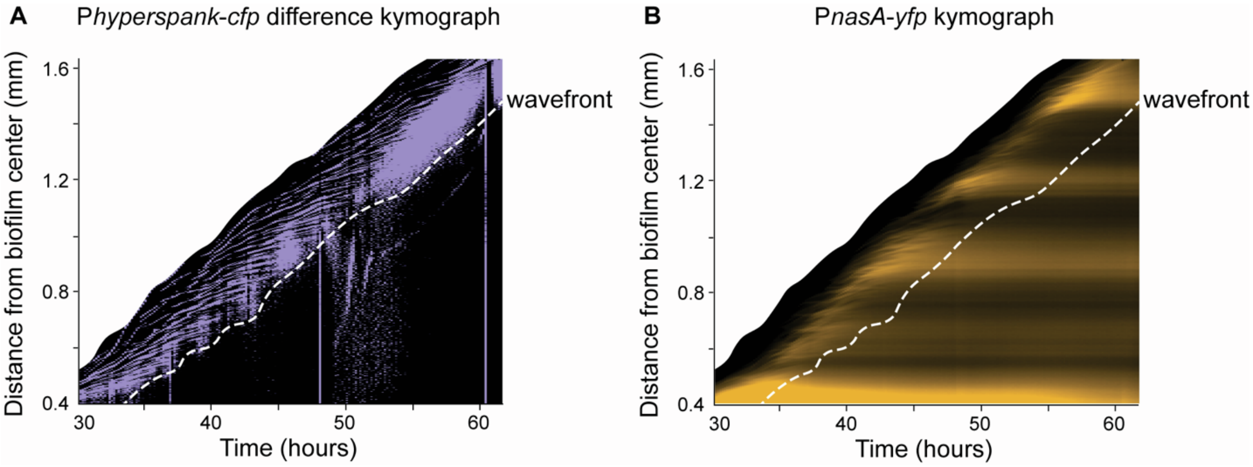

Figure S1. Determination of wavefront position, related to Figure 4.

(A) Kymograph of $P_{hyperspank}$-*cfp* intensity difference between consecutive timepoints. The intensity profile of each timepoint was quantified as described in Fig. 4B. We used binary color code where purple indicates the region above 50% of the peak intensity in each timepoint, and black indicates the region below 50%. The white dashed line indicates the position of the wavefront determined according to the location of 50% of the peak intensity. White regions indicate regions outside of the biofilm determined according to phase-contrast images. Distance indicates radial distance from the center of the biofilm. Time indicates hours after cell loading.

(B) The position of the wavefront from (A) overlaid with P_{nasA}-*yfp* kymograph. White regions indicate regions outside of the biofilm determined according to phase-contrast images. Time indicates hours after cell loading.

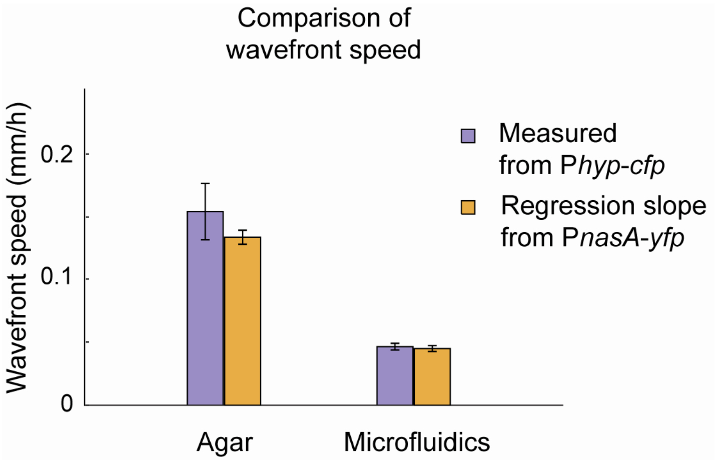

Figure S2. Size and timing of biofilm patterns match the prediction from wavefront speed, related to Figure 4.
Comparison of wavefront speeds directly measured from $P_{hyperspank}$-*cfp* (purple) and the regression slope calculated from P_{nasA}-*yfp* data (orange). We used the distance (S) and period (T) between P_{nasA}-*yfp* rings to calculate the slope of the linear regression, assuming S = vT. For biofilms grown on agar media, measured v = 0.1534 ± 0.0229 mm/h (mean ± 95% confidence interval, n = 12 biofilms), and calculated v' from regression = 0.1332 ± 0.0061 mm/h (mean ± 95% C.I., n = 40 biofilms). For biofilms in the microfluidic device, measured v = 0.0460 ± 0.0028 mm/h (mean ± 95% C.I., n = 31 biofilms), and calculated v' from regression = 0.0449 ± 0.0028 mm/h (mean ± 95% C.I., n = 79 biofilms).

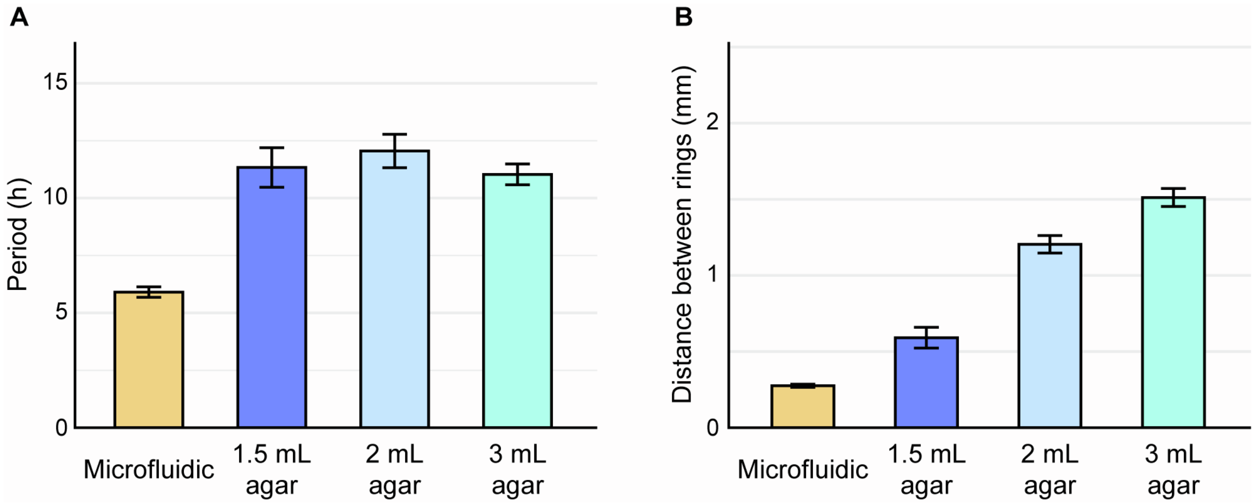

Figure S3. Comparison of period and distance for biofilms in reduced agar conditions, related to Figure 4.

(A) Bar plot showing the oscillation period in different growth conditions. Typical experiments of biofilms grown on agar used 3 mL of media. We decreased the total volume to 2 mL and 1.5 mL. These are the same data points as in Fig. 4I, but plotted in the format of Fig. 4D.

(B) Bar plot showing the distance between rings in different growth conditions. These points are from the same data as panel A.
